# Editorial: Signal Processing for Brain–Computer Interfaces—Special Issue

**DOI:** 10.3390/s24041201

**Published:** 2024-02-12

**Authors:** Noman Naseer, Imran Khan Niazi, Hendrik Santosa

**Affiliations:** 1 Department of Mechatronics Engineering, Air University, Islamabad 44000, Pakistan; 2 Centre for Chiropractic Research, New Zealand College of Chiropractic, Auckland 1060, New Zealand; imran.niazi@nzchiro.co.nz; 3 Department of Radiology, University of Pittsburgh, Pittsburgh, PA 15213, USA; hendrik.santosa@pitt.edu

## 1. Introduction

With the astounding ability to capture a wealth of brain signals, Brain–Computer Interfaces (BCIs) have the potential to revolutionize humans’ quality of life [1] by processing these brain signals for controlling external devices [2]. Being an emerging and innovative field, BCI offers numerous applications in various fields of life, including robotics, education, prosthetics, security and communication technologies [3]. Processing the neuro-physiological signals, a major component of BCI, involves further procedures of (1) noise removal, (2) feature extraction and (3) classification [4]. Pre-processed signals are subject to various noises, including power line noises, physiological noises, motion artifacts and interference noises. These noises can affect the efficiency of the entire BCI procedure. For this reason, noise removal algorithms are utilized for noise removal or reduction [5]. Next, the process of feature extraction starts where algorithms are used to acquire relevant task-based features. This phase acquires data based on spectral and spatial and temporal domains [6]. The last step for signal processing is classification, whereby the acquired and processed features are converted into viable commands, which ultimately control external devices [7]. This Special Issue of *Sensors* focused particularly on these three signal-processing techniques. We invited scientists to share their work conducted on improving performance, information transfer rate, reliability and accuracy of the BCI systems’ signal processing. These works could be based on either non-invasive or invasive techniques, for instance, functional near-infrared spectroscopy (fNIRS), electroencephalography (EEG) and other hybrid brain-imaging techniques. 

## 2. Overview of the Contributions

This issue’s first article uses mental tasks and binary classification tests by employing features of power spectral density (PSD). The authors were able to enhance the classifier’s performance through their peculiar feature set, which can be evidently used in the field of neurophysiology. The second article of this issue applies the P300-BCI Paradigm for controlling home appliances through the brain. The proposed system achieves a high accuracy and shows potential for future application on smart homes. The third article makes use of EEG modality for proposing the multi-kernel temporal and spatial convolution network (MultiT-S ConvNet) based on deep learning and the end-to-end convolutional neural network (ConvNet). The model uses temporal filtering and, thus, can be used to enhance the learning capacity. The fourth article proposes a classification approach using fNIRS-based biomarkers. The data were acquired from both neurotypical and neurodivergent individuals. The four-class classification performances were promising and offered potential for applicability in the field of neuropsychiatry. The fifth article of this issue proposes the design of an unmanned vehicle, using a biomedical sensor, which would be able to monitor health and aid physicians in medical emergencies.

Furthermore, the sixth article of this issue uses fNIRS-based BCI to enhance classification accuracy through Least Absolute Shrinkage and Selection Operator (LASSO) homotopy-based sparse representation classification. This methodology is helpful for rehabilitation purposes in particular. The seventh article conducts brain signal classification acquired through fNIRS signals based on motor execution tasks. The results show enhanced improvement in classification accuracy through deep learning. This classification can be helpful in the field of gait rehabilitation. The eighth article detects ErrPs through EEG in participants with cerebral palsy, amputation or stroke. It also deciphers the discriminative information which is held by different brain regions. This study can be beneficial in formulating adaptive BCIs. The ninth article of this issue utilizes the fNIRS-based approach for upper limb motion detection. The results showed promising accuracy and can be utilized for real-time control. The tenth article works on complicated dexterous task of finger-tapping and acquires data using the fNIRS approach. The classification accuracies appear promising and can be further enriched to generate control commands for BCI application. The eleventh article analyzes the methodologies of 92 studies which utilize fNIRS-EEG-based data and analyses. The review highlights gaps, future directions and potential challenges. 

All of the aforementioned studies accelerate foundational and practical knowledge and application in the field of BCI signal processing. We, the editorial team, appreciate all of these innovative research endeavors and would like to thank the authors for their diligent incorporation of feedback, critical assessment of their work and attentiveness to following the timeline, because of which we have been able to successfully publish this Special Issue. We hope the readers feel inspired by and are able to learn more from the research articles. 

## List of Contributions

1. Rosanne, O.; de Oliveira, A.A.; Falk, T.H. EEG Amplitude Modulation Analysis across Mental Tasks: Towards Improved Active BCIs. *Sensors* **2023**, *23*, 9352.
2. Akram, F.; Alwakeel, A.; Alwakeel, M.; Hijji, M.; Masud, U. A Symbols Based BCI Paradigm for Intelligent Home Control Using P300 Event-Related Potentials. *Sensors* **2022**, *22*, 10000.
3. Emasawas, T.; Morita, T.; Kimura, T.; Fukui, K.; Numao, M. Multi-Kernal Temporal and Spatial Convolution for EEG-Based Emotion Classification. *Sensors* **2022**, *22*, 8250.
4. Erdoğan, S.B.; Yükselen, G. Four-Class Classification of Neuropsychiatric Disorders by Use of Functional Near-Infrared Spectroscopy Derived Biomarkers. *Sensors* **2022**, *22*, 5407.
5. Masud, U.; Saeed, T.; Akram, F.; Malaikah, H.; Akbar, A. Unmanned Aerial Vehicle for Laser Based Biomedical Sensor Development and Examination of Device Trajectory. *Sensors* **2022**, *22*, 3413.
6. Gulraiz, A.; Naseer, N.; Nazeer, H.; Khan, M.J.; Khan, R.A.; Khan, U.S. LASSO Homotopy-Based Sparse Representation Classification for fNIRS-BCI. *Sensors* **2022**, *22*, 2575.
7. Hamid, H.; Naseer, N.; Nazeer, H.; Khan, M.J.; Khan, R.A.; Khan, U.S. Analyzing Classification Performance of fNIRS-BCI for Gait Rehabilitation Using Deep Neural Networks. *Sensors* **2022**, *22*, 1932.
8. Usama, N.; Niazi, I.K.; Dremstrup, K.; Jochumsen, M. Single-Trial Classification of Error-Related Potentials in People with Motor Disabilities: A Study in Cerebral Palsy, Stroke and Amputees. *Sensors* **2022**, *22*, 1676.
9. Sattar, N.Y.; Kausar, Z.; Usama, S.A.; Farooq, U.; Shah, M.F.; Muhammad, S.; Khan, R.; Badran, M. fNIRS-Based Upper Limb Motion Intention Recognition Using an Artificial Neural Network for Transhumeral Amputees. *Sensors* **2022**, *22*, 726.
10. Khan, H.; Noori, F.M.; Yazidi, A.; Zia Uddin, M.; Khan, M.N.A.; Mirtaheri, P. Classification of Individual Finger Movements from Right Hand Using fNIRS Signals. *Sensors* **2021**, *21*, 7943.
11. Li, R.; Yang, D.; Fang, F.; Hong, K.-S.; Reiss, A.L.; Zhang, Y. Concurrent fNIRS and EEG for Brain Function Investigation: A Systematic, Methodology-Focused Review. *Sensors* **2022**, *22*, 5865.
